# Carbon Black Functionalized with Naturally Occurring Compounds in Water Phase for Electrochemical Sensing of Antioxidant Compounds

**DOI:** 10.3390/antiox11102008

**Published:** 2022-10-11

**Authors:** Filippo Silveri, Flavio Della Pelle, Annalisa Scroccarello, Elisabetta Mazzotta, Tiziano Di Giulio, Cosimino Malitesta, Dario Compagnone

**Affiliations:** 1 Faculty of Bioscience and Technology for Food, Agriculture and Environment, University of Teramo, Via R. Balzarini 1, 64100 Teramo, Italy; 2 Laboratorio di Chimica Analitica, Dipartimento di Scienze e Tecnologie Biologiche e Ambientali (Di.S.Te.B.A.), Universitaà del Salento, Via Monteroni, 73100 Lecce, Italy

**Keywords:** nanomaterials, antioxidants, sensors, AuNPs, nanocomposites, green strategy, water dispersed nanomaterials, natural functional compounds

## Abstract

A new sustainable route to nanodispersed and functionalized carbon black in water phase (W-CB) is proposed. The sonochemical strategy exploits ultrasounds to disaggregate the CB, while two selected functional naturally derived compounds, sodium cholate (SC) and rosmarinic acid (RA), act as stabilizing agents ensuring dispersibility in water adhering onto the CB nanoparticles’ surface. Strategically, the CB-RA compound is used to drive the AuNPs self-assembling at room temperature, resulting in a CB surface that is nanodecorated; further, this is achieved without the need for additional reagents. Electrochemical sensors based on the proposed nanomaterials are realized and characterized both morphologically and electrochemically. The W-CBs’ electroanalytical potential is proved in the anodic and cathodic window using caffeic acid (CF) and hydroquinone (HQ), two antioxidant compounds that are significant for food and the environment. For both antioxidants, repeatable (RSD ≤ 3.3%; *n* = 10) and reproducible (RSD ≤ 3.8%; *n* = 3) electroanalysis results were obtained, achieving nanomolar detection limits (CF: 29 nM; HQ: 44 nM). CF and HQ are successfully determined in food and environmental samples (recoveries 97–113%), and also in the presence of other phenolic classes and HQ structural isomers. The water dispersibility of the proposed materials can be an opportunity for (bio) sensor fabrication and sustainable device realization.

## 1. Introduction

Nanotechnology has had a huge impact on the electrochemical and sensoristic scenario, making common the use of carbonaceous materials such as graphene, nanotubes, fullerenes, etc. [1,2,3]; in the last decade, many researchers have moved their attention toward carbon black (CB) due to its excellent proprieties, despite the low cost (EUR ~1 per kg). CB is a material derived from the controlled thermal decomposition in an inert (pyrolysis) or oxygen-depleted (partial combustion) atmosphere of carbon-rich sources, derived mainly from manufacturing byproducts (e.g., vegetable matter, fuel oil, residues from refineries, etc.) [4]. CB appears as a powder formed by micrometric aggregates and agglomerates, where the basic structures are spheroidal particles (CBNPs) with a nano-sized diameter (10–100 nm) containing an amorphous carbon core surrounded by sp^2^ domains [4,5]. To fully exploit CBNPs’ electrochemical features, CB needs to be disaggregated and dispersed before use. A careful procedure leads to a final product with fascinating properties, including high conductivity, fast electron transfer, and a high surface/area ratio that allows the fabrication of high-performing and cost-effective electrochemical devices [5,6,7].

Among the different commercially available CBs, an interesting study by Mazzaracchio et al. [5] demonstrated the superior electron transfer ability of the N220 type. For this reason, this type of CB has been largely employed for electrochemical sensor/device implementation using different approaches: electrode modification via drop casting [7,8,9,10,11], fabrication of transducers via press/thermal transfer [12,13,14,15], or CB inclusion in conductive inks/pastes [16,17]. The classical strategy to prepare/disperse CB for electroanalytical purposes relies on its dispersion in organic solvents. This is needed to gain the strong van der Waals forces among the primary CB particles, which favor aggregation forms in exclusively aqueous systems [4]. This aspect was investigated by Arduini’s group, where the dispersibility of CB N220 in water was poor; further, acetonitrile [9] and mixtures of dimethylformamide [8] were found to be the best solvents. On the other hand, for the transducers fabricated via CB transfer methods, the best solvent was dichloroethane [12,13,14], which allows CB filtration in continuous films. Nevertheless, as they are also prepared in organic solvents, CB dispersions are difficult to homogenize and are often not stable. This affects the electrode/transducer manufacturing and its nanocomposite formation and often does not allow CB functionalization with bio-compounds/receptors in aqueous solutions.

In the last few years, motivated by the ongoing green transition, different approaches have been attempted to improve CB hydrophilicity and avoid organic solvents [4]. Different researchers have employed acidic and/or oxidizing treatments to better enrich the surface chemistry of CB with additional polar groups in order to obtain dispersions in water [18,19,20,21]; however, despite the use of polluting reagents, this has negatively affected the electron transfer ability. A more sustainable route to disperse/exfoliate/stabilize different carbon nanomaterials in water is through sonochemical treatment, which is assisted by surfactants/stabilizing agents. Interestingly, some biological/natural molecules have demonstrated a stabilizing ability towards 1D and 2D nanomaterials of which some are, among others, bile salt sodium cholate [22,23,24,25] and naturally derived polyphenols [26,27,28,29,30]. Recently, our research group proved how the rational selection of the stabilizing agent can give rise to functional nanomaterials with peculiar/additional electrochemical features [30,31]. In this respect, aqueous-dispersed/stabilized CBs have not been deeply investigated [32,33,34]; in particular, despite the exceptional electrochemical features, N220 CB has not yet been studied with this approach. Consequently, nothing has been reported regarding its use in electroanalysis.

Often, the CB electroanalytical features, both for direct sensing and bio-receptor immobilization, are improved by using gold nanoparticles (AuNPs). The CB-AuNPs’ synergy has been exploited to improve electron transfer and the electroanalytical detection of different analytes as phenols [19,35,36,37,38], sugars [36], and pollutant metals [39,40,41]. To form CB-AuNP nanocomposites, different strategies have been proposed. The most-employed strategies include the drop-casting of preformed AuNPs [35,36,37,38,39,40] and the AuNPs’ electrosynthesis onto electrodes previously modified with CB [41,42]. These approaches present some disadvantages, mainly due to the random and/or non-reproducible CB modifications, the tendency to form nanoparticle aggregates, the risk of the formation of metallic micro islands, and the persistence of the metallic precursor. Moreover, the formation of the nanocomposite is strictly related to the use of a pre-existing electrode that has been previously modified with CB, therefore limiting the nanocomposite use in particular configurations. Finally, if a biological element is needed for sensing purposes, it cannot be immobilized directly onto the CB dispersion due to the use of an organic solvent. A few CB-AuNP functionalizations have been attempted in aqueous media [19,43,44]; however, they were based on cumbersome and time-consuming procedures, and additional reagents to trigger the AuNPs’ synthesis were needed.

In this work, CB N220 was nanodispersed and functionalized via a sonochemical approach using sodium cholate (SC)—specifically, a bile salt with inert electrochemistry (CB-SC)—and rosmarinic acid (RA), which is a natural antioxidant that confers a reducing ability (CB-RA). The SC and RA remain anchored onto the CB, thereby allowing their dispersion in water and therefore avoiding CB nanoparticle unit reaggregation. The CB-RA was strategically used to form a nanocomposite via self-assembled gold nanoparticles (CB-RA/AuNPs), driven by the residual phenolic moieties of RA. The physicochemical and morphological features of the W-CBs were carefully studied using SEM, EDX, and XPS. W-CBs were compared with CB, which was dispersed using the conventional approach involving dimethylformamide (CB-DMF). This was conducted to evaluate the impact of the adhered stabilizing agents on the electron transfer and the electroanalytical potentialities. Caffeic acid (CF) and hydroquinone (HQ) were employed to explore the antioxidant sensing abilities of the proposed W-CBs in the anodic and cathodic windows. Finally, the W-CBs’ potentialities to analyze antioxidants in real-world applications were demonstrated analyzing different kinds of food and environmental samples.

## 2. Materials and Methods

### 2.1. Materials, Chemicals, and Samples

Carbon black N220 was obtained from Cabot Corporation (Ravenna, Italy); further, sodium cholate, rosmarinic acid, hydrogen tetrachloroaurate(III), and N-N-dimethylformamide (DMF) were purchased from Sigma-Aldrich (St. Louis, MO, USA). Caffeic acid (CF), hydroquinone (HQ), sodium phosphate monobasic monohydrate, potassium ferrocyanide, potassium ferricyanide, ruthenium hexamine, potassium chloride, methanol, gallic acid, syringic acid, vanillic acid, p-coumaric acid, ferulic acid, catechol, resorcinol, ascorbic acid, citric acid, uric acid, sodium acetate, glucose, fructose, and sucrose were all purchased from Sigma-Aldrich (St. Louis, MO, USA). A total of 10 mM stock solutions of CF and HQ were prepared in methanol and stored at −20 °C in the dark. Milli-Q water (Millipore, Bedford, MA, USA) was used for carbon black dispersion and solution preparations. Samples (thyme, sage, and mint) were purchased from local food shops, and their extraction was carried out as follows: 500 mg of sample was placed in 10 mL of methanol and orbitally stirred (300 rpm) for 1 h in the dark. Then, the sample dispersion was centrifuged at 10,000× *g* for 10 min, and, subsequently, the supernatant was recovered. Finally, the extract was filtered (0.45 µm, PTFE syringe filter) and stored in the dark at −20 °C. Irrigation (Tordino, Teramo, Italy), river mouth (Pescara river, Pescara, Italy), and well (Torre Vecchia Teatina, Chieti, Italy) waters were collected and filtered with a cellulose syringe filter (0.2 μm pore). Before analysis, the food extracts and the environmental water samples were diluted in 0.1 mM of phosphate buffer pH 7 (PB).

### 2.2. Apparatus

The carbon black suspensions were prepared using a probe sonicator SFX550 (wattage output: 550 W; frequency output: 20 kHz) equipped with a 1/8” tapered Microtip (Sonifier^®^ SFX series; Branson Sonic Power Co., Banbury, CO, USA). Centrifugation and the orbital stirring steps were performed with an SL 8R refrigerated centrifuge from Thermo Fisher Scientific (Milano, Italy) and an orbital shaker SSL1 from Stuart Equipment (Belfast, UK), respectively. Electrochemical measures were carried out with a portable Palmsens 4 Potentiostat/Galvanostat/Impedance Analyzer (Palm Instruments BV, Houten, The Netherlands) managed by PS trace software. Screen-printed electrodes (SPE), equipped with a three-electrode configuration (working and counter electrodes of graphite, and a pseudo-reference electrode of Ag/AgCl), were obtained from EcoBioServices (Florence, Italy).

### 2.3. CB Water-Phase Nanodispersion/Functionalization and CB-AuNP Self-Assembling

The CB-SC and CB-RA were prepared as follows: 5 mg of bulk CB N220 powder was placed in a 5 mL water solution containing 1 mg mL^−1^ of stabilizing agent (SC or RA) and then sonicated with a high-performance sonifier equipped with a microtip using a 1 h pulsed program (2 s ON/ 1 s OFF, 30% amplitude); an ice bath was used to keep the temperature below 20 °C. The obtained CB dispersion was centrifuged at 20,000× *g* for 15 min to remove the residual stabilizer; finally, the CB pellet was recovered in 5 mL of water for the CB-SC.

The process for AuNPs self-assembling to form CB-RA/AuNPs was carried out as follows: a CB-RA pellet was resuspended with 4.75 mL of an alkaline water solution (NaOH 0.1 M, pH 13); subsequently, 0.25 mL of 1 mM HAuCl_4_ was added and the suspension was orbitally stirred (300 rpm) for 10 min. Afterward, a centrifugation step at 10,000× *g* (15 min) was employed to discard the HAuCl_4_ excess and purify the obtained CB-RA/AuNPs. The CB-RA/AuNPs pellet was then recovered in 5 mL of water.

CB-DMF, used as a control material, was prepared following the same sonication procedure as the other CBs. However, the CB powder was dispersed in a DMF/H_2_O 1:1 (*v/v*) solution [11]. CB-DMF was not purified from the solvent or through the centrifugation steps.

### 2.4. Physicochemical Characterization

Field emission scanning electron microscopy (SEM) and energy dispersive X-ray spectrometry (EDS) were carried out using a GeminiSEM 500 (Zeiss Co., Oberkrochen, Germany). The carbon black morphological and elemental analyses were performed on uncoated, working electrodes modified with the different CB dispersions—using an acceleration potential of 3 kV and working at a distance of about 3 mm. SEM micrographs were acquired using an In-Lens detector for secondary electrons, and an In-Lens energy selective backscatter (EsB) detector for high-angle backscattered electrons in energy selected mode. EDX was performed using an Oxford Aztec Live Microanalysis system with detector Ultim Max 100 (EDS OXFORD, Buckinghamshire, UK).

X-ray photoelectron spectroscopy (XPS) measurements were recorded with an AXIS ULTRA DLD (Kratos Analytical, Manchester, UK) photoelectron spectrometer, while also using a monochromatic AlKα source (1486.6 eV), operated at 150 W (10 kV, 15 mA). Base pressure in the analysis chamber was set at 5.3 × 10^−9^ Torr. Survey scan spectra were recorded using a pass energy of 160 eV and a 1 eV step. High-resolution spectra were acquired using a pass energy of 20 eV and a 0.1 eV step. The hybrid lens mode was used for all measurements. In each case, the area of analysis was about (700 × 300) μm^2^. During the data acquisition, a system of neutralization of the charge was used. Processing of the spectra was accomplished by the use of CasaXPS Release 2.3.16 software. The binding energy (BE) scale was referenced to the Au 4f7/2 peak at 84.0 eV. For the analysis of high-resolution spectra, all peaks were fitted using the Shirley background and GL(30) line shape (a combination of Gaussian 70% and Lorentzian 30%). For quantitative analysis, the relative sensitivity factors present in the library of CasaXPS for the areas of the signals were used. The graphitic carbon component was fitted with a Voigt line shape exhibiting a higher binding energy tail. Surface charging was corrected considering the binding energy (BE) of C1s (=284.5 eV) of N220 carbon black [5,45].

### 2.5. Electrochemical Characterization and Measurements

Electrochemical features of the proposed materials were studied by drop casting 6 µL of CB suspensions onto the working electrode surface of an SPE. To understand the influence of the stabilizing agent (SC and RA), CB’s electrochemistry was explored via cyclic voltammetry (CV) in PB. The electron transfer capacities of the CBs were investigated with 5 mM [Fe(CN_)6_]^3−/4−^ in 0.1 M KCl, using CV and electrochemical impedance spectroscopy (EIS). EIS experiments were carried out by scanning the frequencies between 10^5^ and 10^−1^ Hz, using a sinusoidal wave of 5 mV amplitude and setting the potential at open circuit; Nyquist plots were then fitted using the Randles-modified equivalent circuit. Caffeic acid (CF) and hydroquinone (HQ) electrochemistry was explored in PB using CV. CF electro-sensing was carried out via chronoamperometry, applying a potential of 0.05 V. HQ electro-sensing was carried out via differential pulse voltammetry (DPV) in the cathodic direction (with a pulse width of 50 ms, modulation amplitude of 50 mV, and a scan rate of 25 mV s^−1^). CB-RA/AuNP electrodes were subjected to two CV scans between −0.35 and 1.35 V (100 mV s^−1^) in 0.05 M H_2_SO_4_ before their use.

## 3. Results and Discussion

The idea behind this work is to exploit naturally derived and biological compounds to nanodisperse and stabilize CB N220 in water phase. This was done to propose a sustainable route for nanomaterials’ preparation and functionalization. Since this has not yet been explored, the idea was to also study the impact of stabilizers on the electrochemical properties and potentialities of CB N220. Further, another aim was to exploit the stabilizer-induced chemistry to drive a nanocomposite self-formation. In brief, this work aims to prove how, using a solvent-free approach and naturally derived functional molecules (including antioxidants), CB/CB-hybrids with captivating features can be easily obtained.

### 3.1. Water-Phase Carbon Black Production and AuNP Self-Assembling

**Scheme 1:**
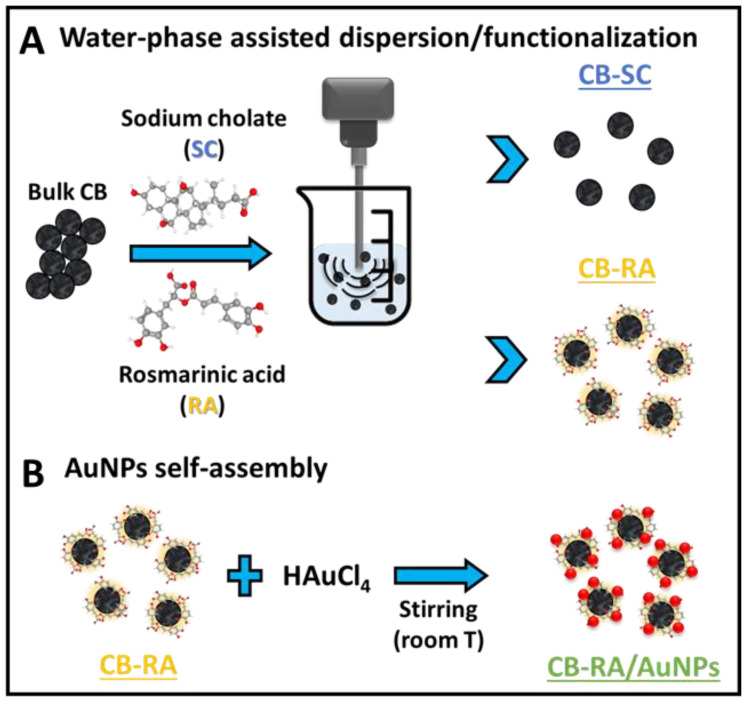
(**A**) Graphic sketch of the CB-SC and CB-RA sonochemical production in water and (**B**) self-assembling of the AuNPs onto the CB-RA colloid.

Among different CB types, CB N220 was selected for its superior electrochemical features; unfortunately, this material has a very low affinity for the water phase. Thus, CB N220 sonochemical nanodispersion/functionalization in water was attempted using different naturally derived molecules as potentially stabilizing agents. Sonochemical treatment of CB N22O (the parameters employed are detailed in Section 2.3) in water was attempted in the presence of different redox-active phenolic compounds, including rosmarinic acid (RA), caffeic acid (CA), tannic acid (TA), and catechin (CT). Further, sodium cholate (SC), a bile salt with a redox-inert behavior, was also tested. Among them, RA and SC were able to give rise to stable black ink-like suspensions, whereas the other compounds returned solutions with visible aggregates and precipitates. Therefore, the obtained CB-SC and CB-RA colloids were selected for subsequent studies; additionally, before their use, the materials were purified removing the excess stabilizing agent. Scheme 1A depicts a graphical sketch of the CB-SC and CB-RA sonochemical production.

Once synthesized and purified, the intimate electrochemistry of the W-CBs was investigated by cyclic voltammetry (CV); to this aim, W-CBs were used to modify SPEs. While no peaks were recorded for CB-SC, the cyclic voltammogram of CB-RA exhibited a well-defined redox pair (Figure S1A), with E_p0_ at 0 V and ΔE of 99 mV. This redox profile is attributable to the catechol moieties of RA, and is characterized by a quasi-reversible electrochemical couple [46,47,48]. The redox couple of CB-RA was then studied to understand if it remain confined to the CB surface. In Figure S1B, CVs in PB performed at different scan rates (5–200 mV s^−1^) are reported. Further, it could be seen that the linear relationship obtained between anodic and cathodic peaks vs. the scan rate (Figure S1C) proved that electron transfer was confined to the surface of the CB-RA modified electrode. The amount of RA attached to the CB surface was estimated using the Sharp approach [31,49], and thus a CB surface coverage (Г) of 1.95 × 10^−4^ mol cm^−2^ was obtained (the equation used is detailed in the caption of Figure S1).

Thus, as illustrated in Scheme 1B, the CB-RA colloid was employed as a platform for the gold nanoparticles’ (AuNPs) self-assembling, exploiting their reducing ability. The CB-RA/AuNPs’ nanocomposite assembling occurs simply by placing the CB-RA colloid in an alkaline medium containing Au(III); the synthesis of AuNPs onto the CB-RA surface occurs at room temperature after just 10 min of shaking. The CB-RA/AuNPs cyclic voltammogram that is reported in Figure S2 suggests the AuNPs’ formation, since an anodic peak at 0.949 V and two consecutive cathodic peaks at 0.727 and 0.551 V are visible [50,51,52]. The CB-AuNPs’ nanocomposite production was also attempted with the use of CB-DMF (see Section 2.3) and CB-SC, with no success. In particular, CB-DMF precipitate in the reaction mix proved the need for a water-dispersed material. CB-SC remains suspended in the reaction mix, but does not allow AuNP formation; this highlights the active role of the RA, both as a stabilizer and reducing agent, given the fact that it is able to drive the Au(III) conversion to Au(0) in order to produce nanoparticles.

Thus, CB-SC and CB-RA/AuNPs were selected for the present study; further, the CB-DMF, since considered the “gold” standard CB N220 preparation strategy for electrochemical purposes, was also used as reference material.

### 3.2. Morphological and Chemical Characterization

**Figure 1:**
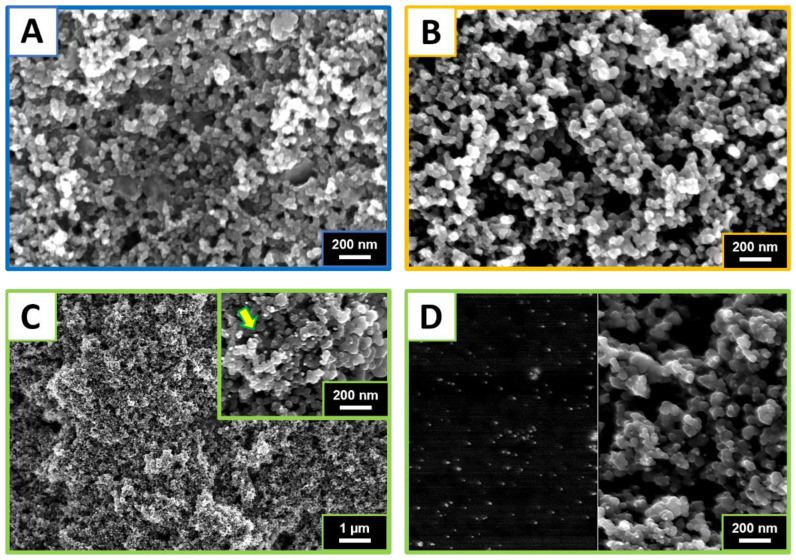
SEM micrographs of (**A**) CB-SC and (**B**) CB-RA, signal acquisition In-Lens Mag 50 Kx. (**C**) SEM micrograph of the CB-RA/AuNPs, signal acquisition In-Lens Mag 10 Kx. Inset: magnification at 50 Kx. The yellow arrow indicates a single AuNP on the CB surface. (**D**) SEM micrographs of the CB-RA/AuNPs at Mag 50 Kx: left signal acquisition EsB; right signal acquisition In-Lens.

Figure 1 reports the SEM micrographs of (A) CB-SC and (B) CB-RA, while Figure S3A depicts that of CB-DMF.

In all cases, CB appears as nanoparticles with a pseudo-spherical shape and a diameter ≤ 40 nm, demonstrating that the procedure does not affect the final CB morphology. Despite the different CBNPs’ phase affinities, the working electrode surface is uniform and completely covered by a 3D CB network. Figure 1C reports the SEM of CB-RA/AuNPs; in this case, in addition to the primary structure formed by the CBNP network, the micrograph reveals the presence of significantly smaller nanoparticles on the CBNPs’ surface. The gold nature of the smaller nanoparticles is confirmed in Figure 1D, where the same micrograph was acquired using two different detectors, i.e., an In-Lens detector (right) and an EsB detector (left) [53]. It can be seen that AuNPs with an average size below 10 nm are deposited on the surface of the CB network. The presence of AuNPs was confirmed by EDS elemental analysis (Figure S3B).

**Figure 2:**
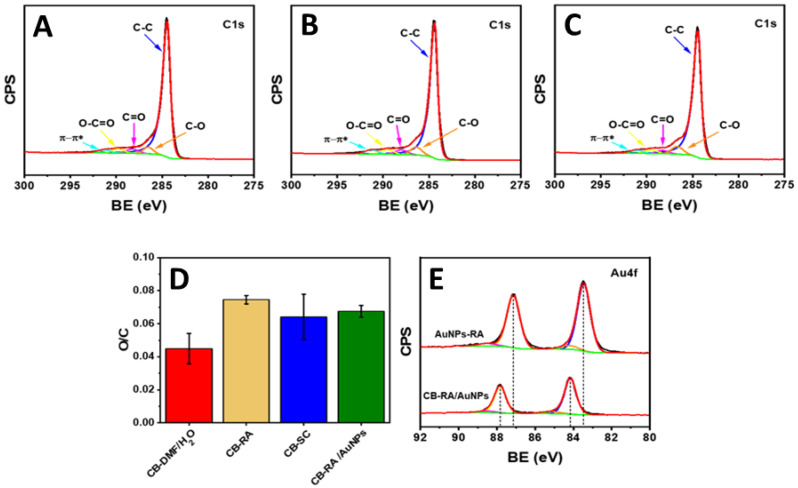
Fitted C1 XPS spectra of (**A**) CB-SC, (**B**) CB-RA, and (**C**) CB-RA/AuNPs. (**D**) O1/C1 (O/C) atomic ratio. (**E**) Fitted Au4f XPS spectra of colloidal AuNPs formed and stabilized using RA (AuNPs-RA), and AuNPs self-assembled onto the CB-RA (CB-RA/AuNPs).

The surface chemistry of CB-SC, CB-RA, and CB-RA/AuNPs was analyzed by XPS; further, the high-resolution C1 signals collected are reported in Figure 2A–C.

The similarity of the carbon spectra of the three samples is clear in each case, with a predominant contribution of the C graphitic component (about 76–86%), as expected. The presence of oxygenated carbon species at a higher binding energy, identified as C-O, C=O, and O-C=O, at about 286.5 eV, 288.3 eV, and 289.5 eV, respectively, is also evident. It is interesting to observe the similarity of the XPS features with respect to CB-DMF (Figure S4A), which was analyzed as a control, as this suggests that the surface chemical composition of CBs obtained by the proposed approach is comparable with that of CB prepared by the conventional method. Nonetheless, some differences emerge in the comparison of the O/C atomic ratios that were estimated on each sample (Figure 2D). While similar values are indeed obtained on CB-SC, CB-RA, and CB-RA/AuNPs, the O/C ratios are higher than that on CB-DMF, possibly representing a further confirmation of the presence of SC and RA as a stabilizing layer on CB particles. The slightly greater O/C ratio in the sample prepared with RA goes in the same direction when being ascribed to the greater amount of oxygenated moieties in RA with respect to SC; this is clearly shown by the fitted C1 signals of the two standards (Figure S4B,C) and the comparison of their O/C ratios (Figure S4D), which are in agreement with stoichiometric ratios. On the other hand, the slight reduction in the O/C ratio in the CB-RA/AuNP sample may be related to the assembly of AuNPs on CB-RA, thereby possibly attenuating the surface signals of the RA shell.

To further investigate the surface chemical composition of the sample prepared by AuNPs’ self-assembly onto CB-RA, Au 4f high-resolution signals were analyzed and compared with the colloidal AuNPs that were formed and stabilized while using RA (AuNPs-RA); the colloidal AuNPs were then synthesized according to Scroccarello et al. [54]. The results reported in Figure 2E reveal that—while Au speciation is the same in the two samples with Au being mostly in its metallic state and with a very minor contribution from oxidized Au at higher binding energy—a remarkable shift of about 0.7 eV toward higher binding energy is recorded on the CB-RA/AuNP sample. This can possibly be ascribed to the quantum size effect of AuNP [55], due to its smaller nanoparticle size when assembled onto CB. Lastly, colloidal AuNP-RA possesses a diameter of about 25 nm.

The observations led to the discovery of a mechanism behind CB-RA stabilization and the reduction in the moieties’ surface functionalization, which can be summarized as follows: (i) the ultrasound induces the untangling of bulk CB aggregates and agglomerates to nanosized particles; (ii) the aromatic skeleton of RA gives rise to π–π interactions within the C-C sp^2^ domains of CB units; (iii) the residual RA polar moieties ensure hydrogen bonds with an aqueous phase; (iv) the reducing power of the RA cathecolic groups [27,31] drive AuNPs’ formation on the CB surface. Similarly, for SC, the steroid ring interacts with the CB surface, and the polar moieties (hydroxylic groups and carboxylic charged group) ensure the nanospheres’ colloidal dispersion in water [22,23]; further, the slightly bent but rigid structure of SC favors the interaction with the spherical shape of the CB nanoparticles [56].

Taking a look at the literature, CB functionalization with gold nanostructures was attempted via different strategies [19,43,44]; nevertheless, AuNPs self-assembling on a CB surface, driven by naturally derived stabilizing agents, has not been reported.

### 3.3. Electrochemical Characterization

**Figure 3:**
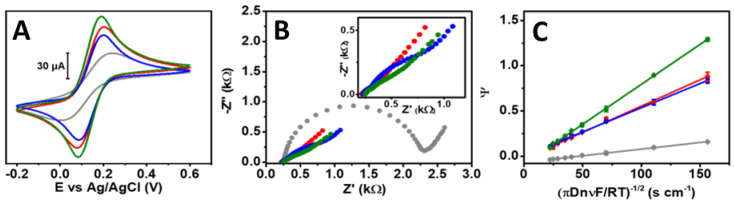
Electrochemical characterization, probe used 5 mM [Fe(CN)_6_]^4−/3−^ in KCl 0.1 M. Sensors legend: bare-SPE (grey), CB-DMF (red), CB-SC (blue), and CB-RA/AuNPs (green). (**A**) Cyclic voltammograms at 25 mV s^−1^. (**B**) Nyquist plot obtained via EIS; inset: magnification at high frequencies. (**C**) Nicholson plot obtained with CVs performed at different scan rates (see Figure S4).

The electrochemical features of the W-CBs were investigated using [Fe(CN)_6_]^4^^−/3^^−^ as an electrochemical probe, while also employing cyclic voltammetry (CV) and electrochemical impedance spectroscopy (EIS). Figure 3A reports the cyclic voltammograms obtained for the W-CBs, in comparison with the CB-DMF and bare SPE.

As expected, CB-DMF (i_pa_ = 80.0 ± 2.1 µA, ΔE = 119 ± 3 mV) leads to a significant improvement of the electron transfer with respect to bare SPE (i_pa_ = 38.5 ± 1.2 µA, ΔE = 217 ± 3 mV), which induces an increase in faradic current and improves the reversibility. Notably, CB-SC was also demonstrated to boost the SPE performance, while having a current intensity (i_pa_ = 70.1 ± 1.4 µA) and reversibility (ΔE = 112 ± 1 mV) that is close to CB-DMF, therefore suggesting similar features for the two CB. The CB-RA/AuNPs gave rise to the best behavior (i_pa_ = 95.2 ± 2.5 µA, ΔE = 102 ± 2 mV), demonstrating higher electron transfer capacities.

The charge transfer resistance (R_ct_) was investigated using EIS, comparing the Nyquist plots of the different CBs studied (Figure 3B); further, the data were fitted using the Randles equivalent circuit. A significantly higher R_ct_, characterized by a wide semicircle, was then obtained for the bare SPE (Rct = 1904 ± 50 Ω). On the other hand, the employment of CB induced a significant decrease in R_ct_, which similarly occurred for CB-SC and CB-DMF (67 ± 6 Ω and 55 ± 4 Ω, respectively). Interestingly, in the CB-SC Nyquist plot, it is possible to notice an additional shoulder in the EIS spectrum at lower frequencies. This behavior suggests an external coating onto the material surface [57,58], attributable in this case to the SC adsorption onto CB. Finally, as expected, the CB-RA/AuNPs exhibited the highest Rct reduction (27 ± 3 Ω), proving the synergistic role of the two nanomaterials in bringing down resistance to charge transfer.

To further explore the electrochemical features, CVs at increasing scan rates were performed for all the materials (Figure S5). In Figure S5A, the linear relationships between the anodic and cathodic peaks, and the square root of the scan rate are reported; the results indicate a diffusion-limited reaction for all the electrodes [59]. Therefore, the electroactive surface area (ECSA) was calculated according to Randles–Sevick’s theory for quasi-reversible systems [60]. Similar ESCAs were obtained for CB-SC and CB-DMF (12.0 ± 0.2 mm^2^ and 13.6 ± 0.4 mm^2^, respectively), which are ~2 folds higher than bare SPE (6.5 ± 0.2 mm^2^), whereas the CB-RA/AuNPs exhibited a significantly higher ECSA of 16.2 ± 0.4 mm^2^.

Finally, the heterogeneous electron transfer constant (k^0^) was estimated, according to the Nicholson method for quasi-reversible systems [61]. The k^0^ was extrapolated from the Nicholson plot (Figure 3C) and the slope of the linear relationship ψ = K^0^πDnFν/RT^−1/2^ was considered, where F is the Faraday constant (sA mol^−1^) and ψ is found with the equation ψ = (−0.6288 + 0.0021X)/(1 − 0.017X), where X is the ΔE (mV) [62]. CB-SC and CB-DMF returned similar K^0^ values (5.3 × 10^−3^ ± 2.4 × 10^−4^ cm s^−1^ and 5.8 × 10^−3^ ± 2.8 × 10^−4^ cm s^−1^, respectively), both significantly higher than bare SPE (1.5 × 10^−3^ ± 1.2 × 10^−4^ cm s^−1^), whereas the greatest K^0^ was obtained for CB-RA/AuNPs (8.8 × 10^−3^ ± 3.4 × 10^−4^ cm s^−1^).

The reproducibility of the CB-SC and CB-RA/AuNPs’ production strategy was estimated by checking the response of five different batches of CB using [Fe(CN)_6_]^4−/3−^. Excellent reproducibility was obtained both for CB-SC (i_pa_ RSD 1.5%; ΔE RSD 0.9%; *n* = 5) and CB-RA/AuNPs (i_pa_ RSD 3.4%; ΔE RSD 3.4%; *n* = 5); the latter result is a particularly high value since it was estimated by considering five complete independent nanocomposite formation procedures.

The stability of CB-SC and CB-RA/AuNPs, as pellets (ready to be resuspended) and on modified electrodes, was then tested. The CB-RA/AuNP pellets (stored at 4 °C) were recorded as stable for 2 months, while CB-SC was stable in this condition for 2 weeks; the stability, in this case, is intended as the stability of the resuspended colloid. It should be pointed out that for CB-DMF, the solution must be used immediately after the dispersion procedure. After SPE modification the storability (at 4 °C) of the modified SPEs was studied for 1 month, and measured every three days [Fe(CN)_6_]^4−/3−^; subsequently, satisfactory results were obtained (i_pa_ RSD 2.3% and 4.2% for CB-SC and CB-RA/AuNPs, respectively).

Overall, CB-SC showed an electron transfer ability that is comparable with the CB-DMF control, as for CB N220, the [Fe(CN)_6_]^4−/3−^ electron transfer is mainly related to edge catalytic and defect sites [63]—where the SC interaction with the CB surface does not affect the electrochemically active sites—while the ability to improve SPE performance confirms the CB-SC nano structuration. On the other hand, CB-RA/AuNPs displayed superior electrokinetic features, thereby proving the synergy between the CB and AuNPs, which positively affects both ECSA and electron transfer.

### 3.4. Electroanalytical Applications

**Figure 4:**
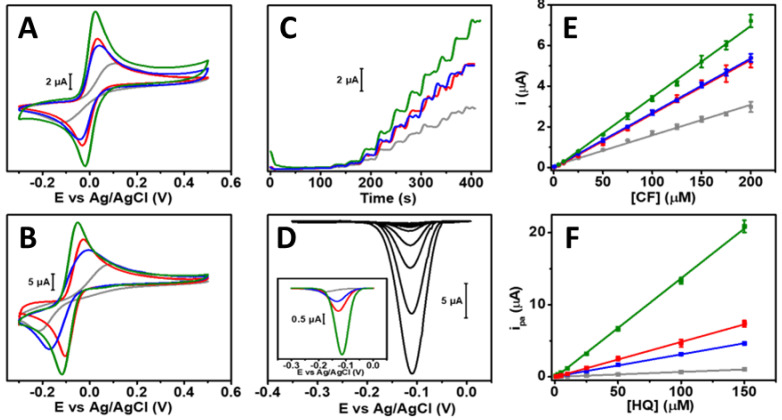
Cyclic voltammograms of (**A**) 500 µM CF and (**B**) 1 mM HQ at 25 mV s^−1^ for the whole set of sensors. (**C**) Chronoamperometry at increasing concentrations of CF (0.5–200 µM) conducted via the whole set of sensors; working potential 0.05 V. (**D**) Differential pulse voltammograms at CB-RA/AuNPs performed with increasing concentrations of HQ (0.5–150 µM); inset: differential pulse voltammograms of 25 µM HQ for the whole set of sensors. Dose-response plots obtained with the whole set of sensors for increasing concentrations of (**E**) CF and (**F**) HQ. Sensor legend: bare-SPE (grey), CB-DMF (red), CB-SC (blue), and CB-RA/AuNPs (green).

The electroanalytical potential of the W-CBs was challenged in the anodic and cathodic windows for the detection of caffeic acid (CF) and hydroquinone (HQ), a food antioxidant compound [64] and an environmental contaminant [65], respectively. CF and HQ electrochemistry were first investigated via CV. Figure 4 demonstrates that the CBs brought a noticeable improvement in the overall electrochemical performance when compared to bare SPE for both CF (Figure 4A) and HQ (Figure 4B). They also possessed higher faradic currents and reversibility, together with favorable detection overpotential shifts.

CB-SC (i_pa_ = 6.9 ± 0.2 µA; ΔE = 78 ± 3 mV) exhibited comparable responses to CB-DMF (i_pa_ = 8.1 ± 0.3 µA; ΔE = 63 ± 4 mV) during CF analysis, whereas a slightly better performance was achieved with CB-DMF for HQ. On the other hand, the CB-RA/AuNPs, for both analytes, had a clear improvement in the reversibility of redox pairs (CF: ΔE = 44 ± 2 mV; HQ: ΔE = 66 ± 4 mV) and a significant increase in the faradic currents (CF: i_pa_ = 11.3 ± 0.5 µA; HQ: i_pa_ = 24.6 ± 0.8 µA).

Chronoamperometry was used for CF determination to increase the sensitivity in order to avoid scanning high overpotentials, while DPV from positive to negative potential was used for HQ to improve the selectivity. Figure S6 depicts the CF hydrodynamic voltammetry performed for all the studied materials. CB-based sensors showed similar behavior, reaching a current plateau at 0.05 V, which was selected as the working potential. Figure 4C shows chronoamperometric measurements performed under increasing concentrations of CF; the respective dose–response curves are reported in Figure 4E. Considering the value of the slopes, the apparent highest sensitivity was obtained for CB-RA/AuNPs (0.0355 ± 0.001 µA uM^−1^); nevertheless, CB-SC—despite the lower slope (0.0268 ± 0.001 µA uM^−1^)—gave the best limit of detection, i.e., LOD = 29 nM (linear range 0.5–200 µM; i (µA) = 0.0268 (µM) + 0.0106; R^2^ = 0.9999). Further, LOD was calculated as 3× the standard deviation of the intercept/slope. This behavior is attributable to the CB-RA/AuNPs’ higher capacitive current for CF determination, which negatively affected the analytical performance, resulting in lower LOD and a shorter linear range. Thus, CB-SC was selected for CF determination.

Figure 4D reports the DPV for HQ detection performed with all the studied materials. In this case, a significantly higher reduction current at a more positive overpotential was obtained for the CB-RA/AuNPs, followed by CB-DMF and CB-SC; the bare SPE, however, had a significantly lower response. Figure 4D and Figure S7 show DPVs performed with CB-RA/AuNPs and the other tested materials under increasing concentrations of HQ. As displayed from the dose–response slopes that were extrapolated for each sensor (plots in Figure 4F), the previous trend was confirmed; the nanocomposite has a sensitivity ~3–4 folds higher than CB-SC and CB-DMF, and ~20 folds higher compared to the unmodified SPE. CB-RA/AuNPs’ response was linearly between 0.5–150 µM (i (µA) = 0.138 (µ–) − 0.109; R^2^ = 0.9993), and a LOD of 44 nM was obtained. It must be noted, that carbonaceous nanomaterials’ decoration with AuNPs has been widely employed to improve HQ determination performance [19,35,66,67,68].

The fouling resistance of the selected sensors was tested by performing 10 consecutive measures with CF and HQ; repeatable signals were obtained for both CB-SC (CF: RSD 3.3%, *n* = 10) and CB-RA/AuNPs (HQ: RSD 2.9%, *n* = 10). Finally, the reproducibility was assessed by performing three calibration curves using different electrodes, the similar slopes obtained for CF (CB-SC slope RSD < 3.3%; *n* = 3) and HQ (CB-SC slope RSD < 3.8% *n* = 3) demonstrated a satisfactory inter-sensors precision.

When considering the literature, CF and HQ determination can be considered a model determination of two antioxidant analytes with well-known electrochemical behavior, and, for this reason, was performed by using a plethora of nanomaterials. CF determination via chronoamperometry is mainly employed for biosensing purposes [69], whereas works involving direct amperometric sensing of CF are summarized in Table S1. Table S2 reports the main analytical findings of works concerning voltammetric HQ electrosensing using nanocomposite-based sensors. Despite the sustainable synthesis route followed in the present work, both CB-SC and CB-RA/AuNPs have allowed us to achieve a better, or comparable, analytical performance when compared with the literature, which also proves the antifouling features.

### 3.5. Sample Analysis and Selectivity

The suitability of W-CB sensors for real-life applications was investigated using food and environmental samples; three commercial spices were collected (thyme, sage, and mint) and analyzed for CF determination, while environmental waters (irrigation, river mouth, and well) were employed for HQ analysis. Phenolic compounds in vegetable matrices are present in complex mixtures, composed of different classes that can be grouped in terms of reactivity; therefore, the CB-SC electrochemical signal was attributed to CF equivalents present in the samples, which include o-diphenolic structures that can be oxidizable at the lowest potentials [24,62]. On the other hand, HQ can be considered a contaminant, and its presence in environmental waters should be found in trace amounts [65]. The accuracy of the proposed W-CB sensors in real matrices was assessed by spiking samples at three concentration levels and quantifying them using the standard addition method. Table 1 lists the full set of the obtained data, while Figure S8 reports an example of chronoamperometric and voltametric analysis carried out with a plant extract and environmental water.

The CB-SC and CB-RA/AuNPs allowed us to obtain satisfactory recoveries for CF (98–113%) and HQ (97–104%), respectively. Additionally, for all the samples assessed, reproducible results were obtained (CF RSD ≤ 5.7%, HQ RSD ≤ 6.0%; *n* = 3).

**Figure 5:**
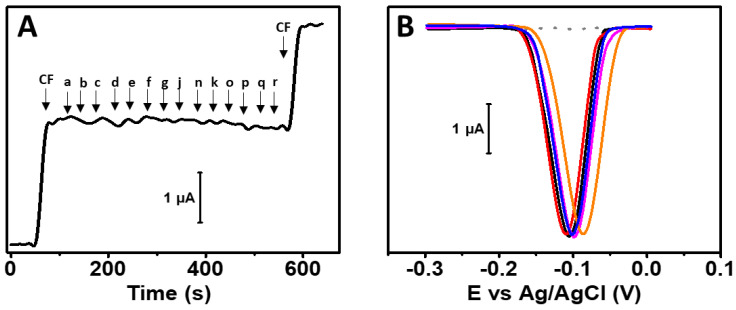
(**A**) Chronoamperometry response of 100 µM CF at CB-SC before and after the addition of potentially interfering species. Spiked compound legend: 100 µM caffeic acid (CF), 10 µM gallic acid (a), 10 µM vanillic acid (b), 10 µM ferulic acid (c), 10 µM syringic acid (d), 10 µM p-coumaric acid (e), 1 mM glucose (f), 1 mM fructose (g), 1 mM sucrose (h), 10 µM citric acid (i), 10 µM acetic acid (j), 10 µM ascorbic acid (k), 1 mM K^+^ (l), 1 mM Na^+^ (m), 1 mM Mg^2+^ (n), 1 mM Cl^−^ (o), 1 mM PO_4_^−^ (p), 1 mM NO_2_^−^ (q) 1 mM NO_3_^−^ (r). (**B**) Differential pulse voltammograms of 50 µM HQ in the absence and presence of potentially interfering species. Voltammograms legend: 50 µM HQ (black curve); 50 µM HQ in presence of 20 µM catechol, 20 µM resorcinol, 20 µM glucose, 20 µM fructose (red curve); 50 µM HQ in presence of 20 µM ascorbic acid, 100 µM acetic acid, 100 µM citric acid, 20 µM uric acid (blue curve); 50 µM HQ in presence of 1 mM Cl^−^, 1 mM Na^+^, 1 mM Mg^2+^, 1 mM Ca^2+^ (magenta curve); 50 µM HQ in presence of 200 µM NO_3_^−^, 200 µM NO_2_^−^, 1 mM PO_4_^−^, 100 µM CO_3_^2−^ (orange curve).

The selectivity of W-CB sensors was evaluated, and an analysis of the electrochemical response of CF and HQ, in the presence of potentially interfering species, was conducted. Figure 5A reports the chronoamperometric response of CF at the CB-SC electrode, before and after spiking different potential interferences. Further, several compounds were tested, including phenolic structures with different hydroxyl moieties, sugars, organic acids, and inorganic ions (see the full list in the caption below).

The tested compounds made no significant alterations to the amperometric response; furthermore, at the end of the test, the current intensity returned by CF was similar to the starting current intensity. Notably, the very low overpotential (+0.05 V) CB-SC result was selective towards CF (o-diphenols) even in the presence of other phenolic classes such as trihydroxy-phenols, mono-phenols, and phenols with methoxy moieties.

The CB-RA/AuNPs’ selectivity toward HQ detection was tested, and HQ DPV scans in the presence of potentially interfering compounds were carried out. Figure 5B displays the measures performed with CB-RA/AuNPs in the presence of HQ isomers, sugars, organic molecules, and inorganic ions (please see the complete list in the caption above). A satisfactory selectivity was achieved since the response of CB-RA/AuNPs to HQ was not significantly affected by the presence of the tested compounds. It is interesting to note how the HQ signal is not perturbed by the presence of the structural isomers catechol and resorcinol, which often coexist in environmental samples. Other organic molecules, such as sugars or ascorbic acid, did not interfere as they were electroactive in the anodic domain. Overall, the obtained results demonstrate the exploitability of the proposed W-CB sensors not only in model solutions, but also for real applications.

## 4. Conclusions

In this work, a new sustainable route to nanodispersed/stabilized and functionalized carbon black N220 in the water phase (W-CB) for electroanalytical purposes is presented, with particular attention paid to antioxidant compound analysis. The sonochemical strategy exploits ultrasound to disaggregate the CB nanoparticles, while the two functional, naturally derived compounds (sodium cholate and rosmarinic acid) act as stabilizing agents, thereby ensuring dispersibility in water and adherence to the surface of CB nanoparticles. Strategically, CB-RA was used to drive AuNPs’ self-assembling at room temperature, resulting in a CB surface nanodecorated with gold; this was achieved without the need for additional reagents, and the nanocomposite was also water stable. The W-CBs showed remarkable electron transfer, and the CB-RA/AuNPs’ nanocomposite boosted the electrochemical properties of CB, resulting in the most effective material. On the other hand, CB-SC showed properties similar to CB when prepared in the standard way in DMF, resulting in a green alternative. The W-CBs proved their electroanalytical potential in model solutions and real samples in the anodic and cathodic window, which was demonstrated via caffeic acid and hydroquinone determination down to nanomolar levels. In summary, W-CBs are new nanomaterials used for the purposes of electrosensing, and their sustainable production is solvent-free and provides a reliable and greener alternative for the preparation of nano CB and AuNP-nanodecorated CB. Notably, the proposed materials are extremely prone to phenolic antioxidants analysis; on the other hand, a functional role was demonstrated for another antioxidant, rosmarinic acid, which acts as a functional molecule. The water dispersibility of the proposed materials can be an opportunity for in-solution functionalization of CB/CB-AuNPs with biological elements and receptors, thus also opening up new paths for the development of such devices and biosensors.

## Figures and Tables

**Table 1:** CF_eq_ and HQ determination in samples, data expressed as mean value (*n* = 3).

Caffeic Acid Equivalents					Hydroquinone				
Samples	Added	Found	Recovery	RSD	Samples	Added	Found	Recovery	RSD
	(µM)	(µM)	(%)	(%)		(µM)	(µM)	(%)	(%)
Thyme	-	41.2	-	5.1	Irrigation	-	-	-	-
	10	51.6	104.1	4.0		0.5	0.5	101.6	5.6
	20	61.4	101.3	5.7		5	5.1	101.4	4.2
	30	72.1	102.9	2.3		10	10.4	103.6	3.5
Sage	-	96.0	-	2.9	River mouth	-	-	-	-
	10	107.0	110.2	3.3		0.5	05	97.6	4.8
	20	115.5	97.5	3.2		5	5.1	102.9	2.9
	30	125.7	99.0	4.4		10	10.2	101.9	3.7
Mint	-	105.5	-	4.1	Well	-	-	-	-
	10	116.8	112.8	5.4		0.5	0.5	97.1	6.0
	20	127.3	108.9	4.3		5	4.8	97.5	3.1
	30	135.4	99.7	3.4		10	9.7	97.0	5.9

## Data Availability

All data are included within the article and supplementary.

## Supplementary Materials

The following supporting information can be downloaded at: https://www.mdpi.com/article/10.3390/antiox11102008/s1. Figure S1: (**A**) CB-RA cyclic voltammogram in PB, (**B**) CB-RA cyclic voltammograms in PB performed at increasing scan rates, (**C**) i_pa_ and i_pc_ linear relationship. Figure S2: cyclic voltammogram of CB-RA/AuNPs in H_2_SO_4_. Figure S3: (**A**) SEM micrographs of CB-DMF, (**B**) EDS spectrum of the CB-RA/AuNPs. Figure S4: C1 XPS spectra of (**A**) CB-DMF and of the (**B**) SC and (**C**) RA standards, (**D**) O/C atomic ratios of SC and RA standards. Figure S5: (**A**) linear relationship between i_pa_ and i_pc_ and the square root of the scan rate for the whole set of sensors using [Fe(CN)_6_]^4−/3−^, cyclic voltammograms of [Fe(CN)_6_]^4−/3−^ at increasing scan rates for (**B**) CB-SC and (**C**) CB-RA/AuNP. Figure S6: Hydrodynamic voltammetry of CF performed with the whole set of sensors. Figure S7: DPV of increasing concentrations of HQ, performed at the (**A**) bare-SPE, (**B**) CB-DMF, and (**C**) CB-SC. Figure S8: (**A**) Chronoamperometry of mint sample spiked with CF, (**B**) DPV of irrigation water fortified with HQ. Table S1: Literature overview of nanomaterial-based chronoamperometric sensors for CF determination. Table S2: Literature overview on nanocomposite-based voltammetric sensors for HQ determination.
