# Supplementary material for: Carbon Black Functionalized with Naturally Occurring Compounds in Water Phase for Electrochemical Sensing of Antioxidant Compounds
Source: Antioxidants (Basel). 2022 Oct 11;11(10):2008. doi: 10.3390/antiox11102008 (PMC9598705; doi:10.3390/antiox11102008)
Supplement: Supplementary file 1 [file antioxidants-11-02008-s001.zip › antioxidants-1925281-supplementary.pdf]

SUPPORTING MATERIAL

Carbon black functionalized with naturally occurring compounds in water phase for electrochemical sensing of antioxidant compounds

Filippo Silveri[a], Flavio Della Pelle[a,*], Annalisa Scroccarello[a], Elisabetta Mazzotta[b], Tiziano Di Giulio[b], Cosimino Malitesta[b], Dario Compagnone[a,**].

[a]Faculty of Bioscience and Technology for Food, Agriculture and Environment, University of Teramo, Campus "Aurelio Saliceti" Via R. Balzarini 1, 64100, Teramo, Italy

[b]Laboratorio di Chimica Analitica, Dipartimento di Scienze e Tecnologie Biologiche e Ambientali (Di.S.Te.B.A.), Università del Salento, via Monteroni, Lecce 73100, Italy

Corresponding Authors:

* e-mail: fdellapelle@unite.it (F. Della Pelle); telephone number: +39 0861-266948

** e-mail: dcompagnone (D. Compagnone); telephone number: +39 0861-266942

Table of Contents

SM 1. Supplementary figures **3**

SM 2. Supplementary tables **11**

SM 3. Supplementary References **12**

M 2. Supplementary figures

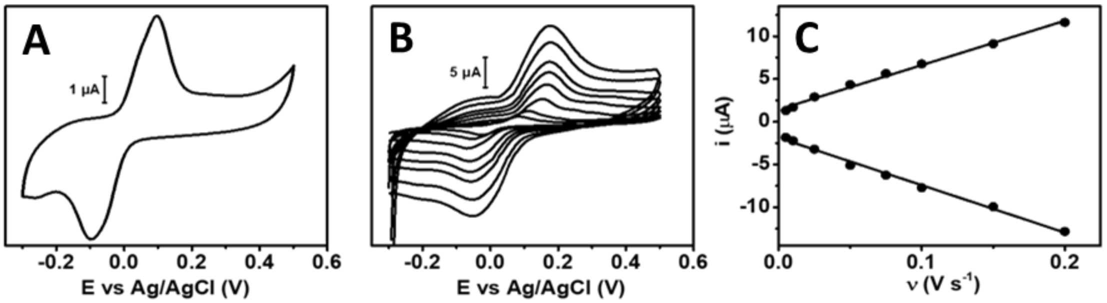

Figure S1. (**A**) CB-RA cyclic voltammogram in PB at 25 mV s^{-1}. (**B**) CB-RA cyclic voltammograms performed at increasing scan rates (from 5 to 200 mV s^{-1}). (**C**) Linear relationship (black lines) obtained for i_{pa} and i_{pc} under increasing scan rates (from 5 to 200 mV s^{-1}). Equation used to estimate the surface coverage of the CB-RA: $\Gamma=(ip_a 4RT)/n^2F^2Av$, where R is the gas constant (8.314 J K^{-1} mol $^{-1}$), F is the Faraday's constant (96500 C mol^{-1}), T is the temperature (K), n is the number of electrons involved, A the electrode geometrical area (cm^2), and ip_a/v is the slope of anodic peaks vs scan rates (A/v s^{-1}).

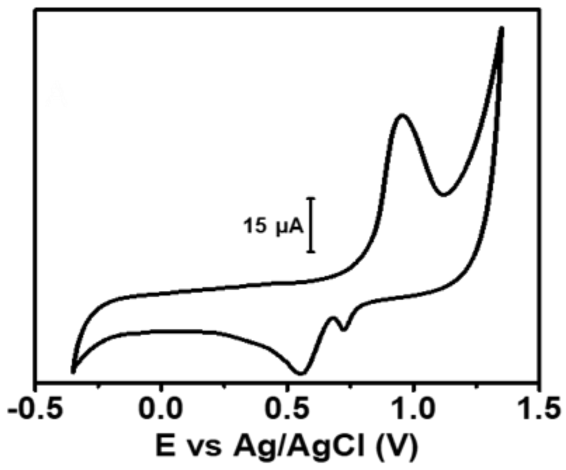

Figure S2. Cyclic voltammogram of the CB-RA/AuNPs obtained in 0.05 M H_2SO_4, scan rate 50 mV s^{-1}.

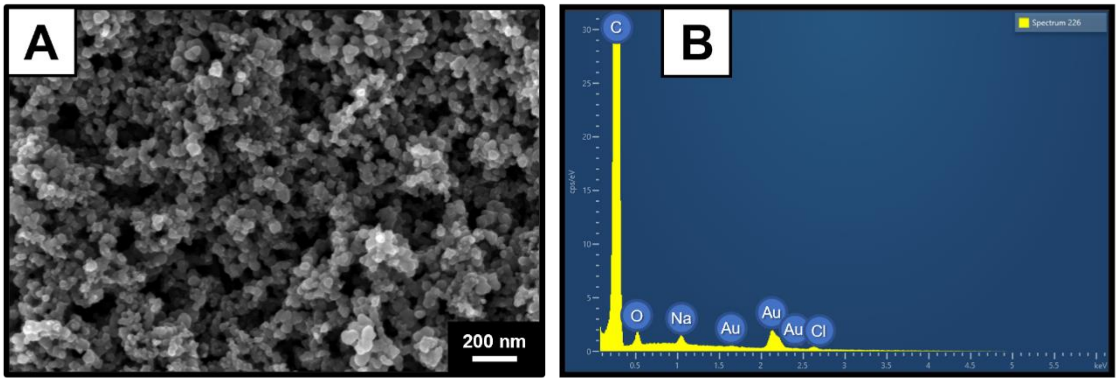

Figure S3. SEM micrographs of the (**A**) CB-DMF signal acquisition In-Lens Mag 50 Kx. (**B**) EDS spectrum of the CB-RA/AuNPs.

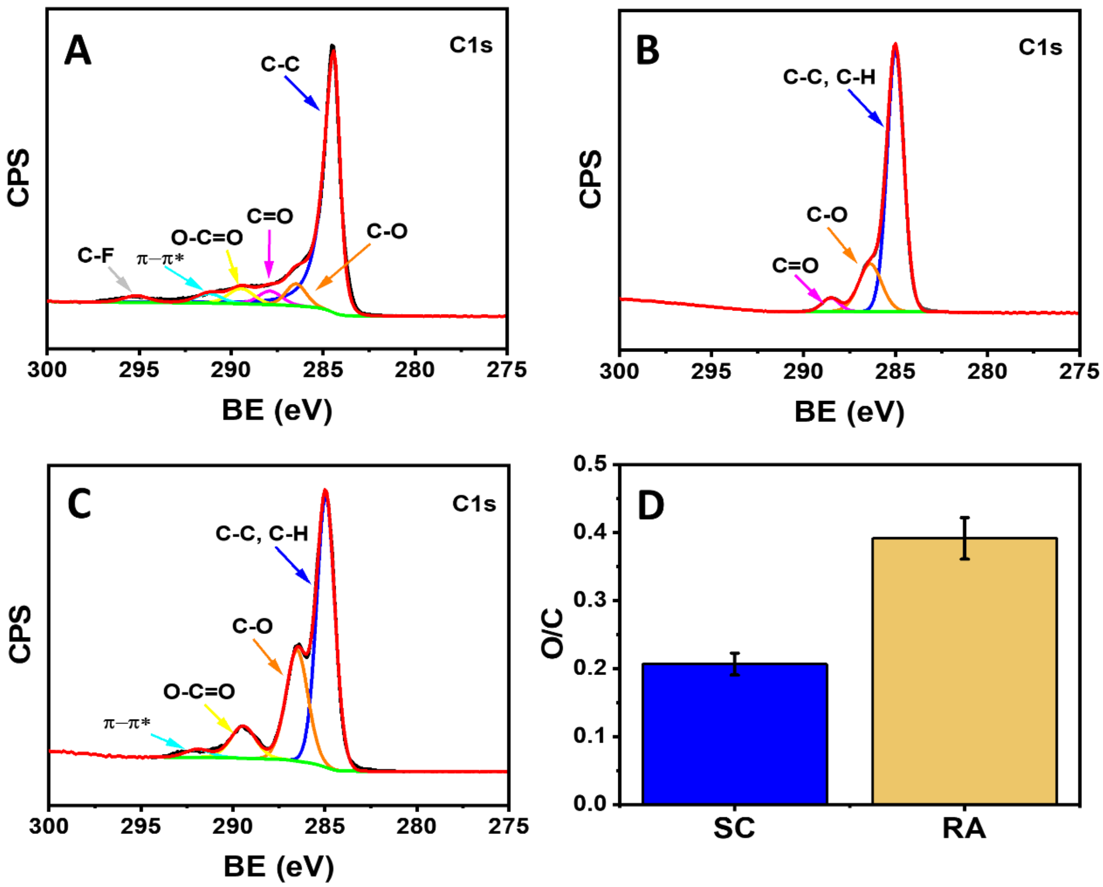

Figure S4. Fitted C1s XPS spectra of the (**A**) CB-DMF and of the (**B**) SC and (**C**) RA standards. (**D**) O/C atomic ratios of SC and RA standards.

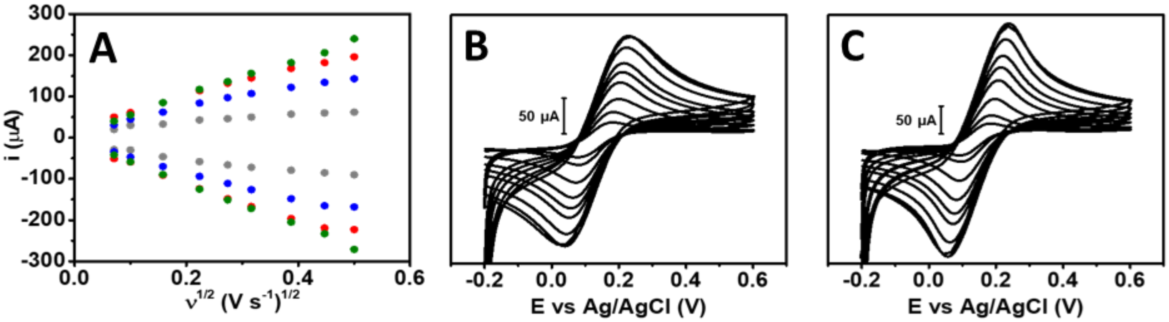

Figure S5. (**A**) Linear relationship between i_{pa} and i_{pc} and the square root of the scan rate obtained for the whole set of sensors. Sensor legend: bare-SPE (grey), CB-DMF (red), CB-SC (blue), CB-RA/AuNPs (green). Cyclic voltammograms of 5 mM $[Fe(CN)_6]^{4-/3-}$ in KCl 0.1 M performed at scan rates from 5 to 200 mV s^{-1} at the (**B**) CB-SC and (**C**) CB-RA/AuNPs.

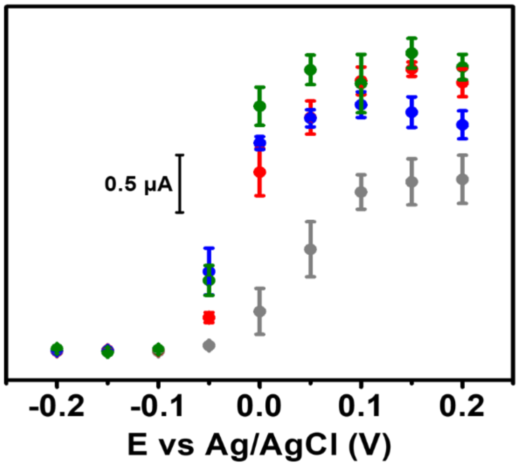

Figure S6. Hydrodynamic voltammetry performed with 100 µM CF in PB at the different sensors. Sensor legend: bare-SPE (grey), CB-DMF (red), CB-SC (blue), CB-RA/AuNPs (green).

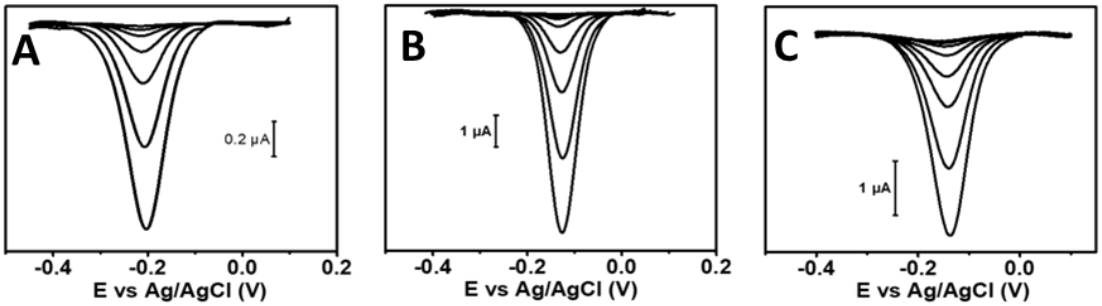

Figure S7. Differential pulse voltammograms of increasing concentrations of HQ (0.5-150 µM) in PB, analysis performed at the (**A**) bare-SPE, (**B**) CB-DMF and (**C**) CB-SC.

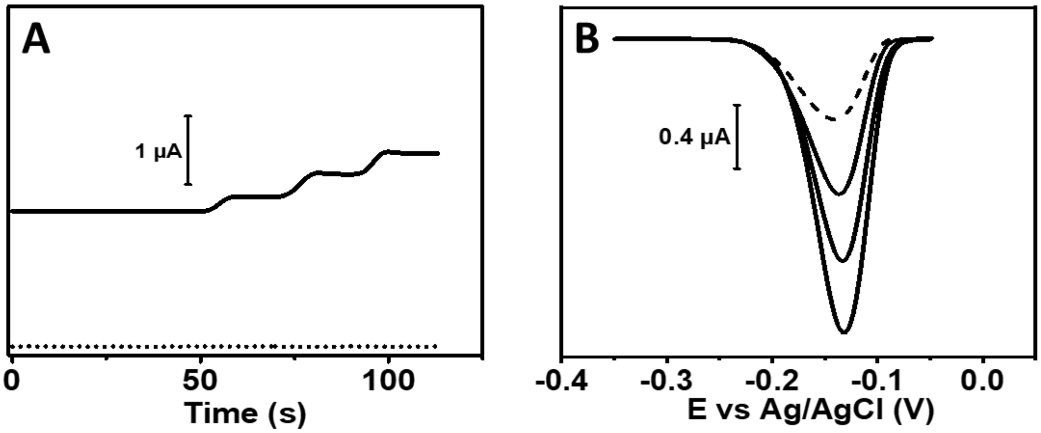

Figure S8. (**A**) Continuous line: chronoamperometry of mint sample in PB spiked with 10, 20, and 30 µM of CF. Dotted line: chronoamperometry of only PB, used as blank. Measures performed using a working potential of 0.05 V. (**B**) Dashed curve: differential pulse voltammogram of irrigation water fortified with 10 µM HQ. Solid curves: differential pulse voltammograms of the fortified irrigation water sample spiked with 10, 20, and 30 µM HQ.

SM 2. Supplementary tables

Table S1. Employed electrodes, materials, overpotential applied, and main analytical features of nanomaterials-based chronoamperometric sensors for CF determination.

Electrode*	Material**	Potential (V)	LOD (nM)	L.R. (µM)	Ref
GCE	CRGO	0.20	2	0.01-800	[1]
GCE	3DG-MWCNTs	0.20	18	0.2-174	[2]
GCE	$CuZnO_x$-MWCNTs	0.20	155	10-100	[3]
GCE	Cu-MOF	0.32	40	0.05-230	[4]
SPE	CB-SC	0.05	29	0.5-200	This work

*GCE: glassy carbon electrode. SPE: screen printed electrode. **CRGO: chemically reduced graphene oxide. 3D-MWCNTs: three-dimensional graphene-multiwalled carbon nanotubes. $CuZnO_x$/MWCNTs: CuO-ZnO-multiwalled carbon nanotubes. Cu-MOF: CuO nanoparticles MOF (metal organic framework) mediated synthetized. CB-SC: carbon black-sodium cholate.

Table S2. Employed electrodes, materials, electrochemical technique, and main analytical features of nanocomposite-based voltammetric sensors for HQ determination.

Electrode*	Material**	Technique***	LOD (nM)	L.R. (µM)	Ref
GCE	Pt-Gr	DPV red	6000	20-145	[5]
CILE	AuNPs-Gr	DPV red	18	0.06-800	[6]
GCE	MET-AuNPs	DPV red	120	8-400	[7]
GCE	RGO-$[Cu(en)_2]^{2+}$	DPV ox	25	0.1-104	[8]
GCE	EGr-Au	SWV ox	100	0.3-100	[9]
GCE	Au@Pd	DPV ox	630	4-5000	[10]
GCE	PNMTh-RGO	DPV ox	750	1-1000	[11]
GCE	N-RGO-$SrZrO_3$	SWV ox	610	2.5-2500	[12]
SPE	CB-RA/AuNPs	DPV red	44	0.5-150	This work

*GCE: glassy carbon electrode. CILE: carbon ionic liquid electrode. SPE: screen printed electrode. **Pt-graphene: platinum graphene hybrid. AuNPs-Gr: gold nanoparticles and graphene. MET-AuNPs: methionine-gold nanoparticles. RGO-$[Cu(en)_2]^{2+}$: reduced graphene oxide decorated copper(II) ethylenediamine complex. EGr: graphene nanosheet-gold nanoparticles. Au@Pd: gold-palladium core shell nanocomposite. PNMTh-RGO: poly(N-methylthionine)-reduced graphene oxide. N-RGO-$SrZrO_3$: nitrogen doped graphene oxide- strontium zirconate. CB-RA/AuNPs: carbon black-rosmarinic acid-gold nanoparticles. *** DPV: differential pulsed voltammetry. SWV: square wave voltammetry.

SM 3. Supplementary References

1. Ezhil Vilian, A.T.; Chen, S.M.; Chen, Y.H.; Ajmal Ali, M.; Al-Hemaid, F.M.A. An Electrocatalytic Oxidation and Voltammetric Method Using a Chemically Reduced Graphene Oxide Film for the Determination of Caffeic Acid. *J Colloid Interface Sci* **2014**, *423*, 33–40, doi:10.1016/j.jcis.2014.02.016.
2. Sakthinathan, S.; Kubendhiran, S.; Chen, S.M. Hydrothermal Synthesis of Three Dimensional Graphene-Multiwalled Carbon Nanotube Nanocomposite for Enhanced Electro Catalytic Oxidation of Caffeic Acid. *Electroanalysis* **2017**, *29*, 1103–1112, doi:10.1002/elan.201600687.
3. Xie, A.; Wang, H.; Zhu, J.; Chang, J.; Gu, L.; Liu, C.; Yang, Y.; Ren, Y.; Luo, S. A Caffeic Acid Sensor Based on CuZnOx/MWCNTs Composite Modified Electrode. *Microchemical Journal* **2021**, *161*, doi:10.1016/j.microc.2020.105786.
4. Venkadesh, A.; Mathiyarasu, J.; Radhakrishnan, S. MOF Mediated Synthesis of Porous Copper Oxide and Their Electrochemical Sensing of Caffeic Acid in Caffeinated Drinks. *Inorg Chem Commun* **2021**, *128*, doi:10.1016/j.inoche.2021.108573.
5. Li, J.; Liu, C.Y.; Cheng, C. Electrochemical Detection of Hydroquinone by Graphene and Pt-Graphene Hybrid Material Synthesized through a Microwave-Assisted Chemical Reduction Process. *Electrochim Acta* **2011**, *56*, 2712–2716, doi:10.1016/j.electacta.2010.12.046.
6. Hu, S.; Wang, Y.; Wang, X.; Xu, L.; Xiang, J.; Sun, W. Electrochemical Detection of Hydroquinone with a Gold Nanoparticle and Graphene Modified Carbon Ionic Liquid Electrode. *Sens Actuators B Chem* **2012**, *168*, 27–33, doi:10.1016/j.snb.2011.12.108.
7. He, J.; Song, Z.; Zhang, S.; Wang, L.; Zhang, Y.; Qiu, R. Methionine – Au Nanoparticle Modified Glassy Carbon Electrode: A Novel Platform for Electrochemical Detection of Hydroquinone. *Medziagotyra* **2014**, *20*, 381–386, doi:10.5755/j01.ms.20.4.6477.
8. Sakthinathan, S.; Kokulnathan, T.; Chen, S.M.; Karthik, R.; Chiu, T.W. Ecofriendly Preparation of Graphene Sheets Decorated with an Ethylenediamine Copper(II) Complex Composite Modified Electrode for the Selective Detection of Hydroquinone in Water. *Inorg Chem Front* **2018**, *5*, 490–500, doi:10.1039/c7qi00640c.
9. Pogacean, F.; Coroş, M.; Magerusan, L.; Rosu, M.-C.; Socaci, C.; Gergely, S.; Stefan-van Staden, R.-I.; Moldovan, M.; Sarosi, C.; Pruneanu, S. Sensitive Detection of Hydroquinone Using Exfoliated Graphene-Au/Glassy Carbon Modified Electrode. *Nanotechnology* **2017**, *29*.
10. Chen, T.; Xu, J.; Arsalan, M.; Sheng, Q.; Zheng, J.; Cao, W.; Yue, T. Controlled Synthesis of Au@Pd Core-Shell Nanocomposites and Their Application for Electrochemical Sensing of Hydroquinone. *Talanta* **2019**, *198*, 78–85, doi:10.1016/j.talanta.2019.01.094.
11. Liu, Y.; Song, N.; Ma, Z.; Zhou, K.; Gan, Z.; Gao, Y.; Tang, S.; Chen, C. Synthesis of a Poly(N-Methylthionine)/Reduced Graphene Oxide Nanocomposite for the Detection of

Hydroquinone. *Mater Chem Phys* **2019**, *223*, 548–556, doi:10.1016/j.matchemphys.2018.11.045.

12. Ahmad, K.; Kumar, P.; Mobin, S.M. A Highly Sensitive and Selective Hydroquinone Sensor Based on a Newly Designed N-RGO/SrZrO3 Composite. *Nanoscale Adv* **2020**, *2*, 502–511, doi:10.1039/c9na00573k.
